# Identification of Key Gene Networks Controlling Soluble Sugar and Organic Acid Metabolism During Oriental Melon Fruit Development by Integrated Analysis of Metabolic and Transcriptomic Analyses

**DOI:** 10.3389/fpls.2022.830517

**Published:** 2022-05-12

**Authors:** Hong Cheng, Weiping Kong, Taoxia Tang, Kaili Ren, Kaili Zhang, Huxia Wei, Tao Lin

**Affiliations:** ^1^Vegetable Research Institute, Gansu Academy of Agricultural Sciences, Lanzhou, China; ^2^College of Horticulture, China Agricultural University, Beijing, China

**Keywords:** oriental melon, metabolome, transcriptome, fruit, soluble sugar, organic acid

## Abstract

Oriental melon (*Cucumis melo* var. *acidulus*) is one of the most economically important fruit crops worldwide. To elucidate the molecular basis related to soluble sugar and organic acid metabolism in the fruits of two oriental melon cultivars with different sweetness, we performed integrated metabolomic and transcriptomic analyses of the fruits of ‘Tianbao’ (A) with high sweetness and ‘Xiaocuigua’ (B) with low sweetness at different ripening stages. The high accumulation of sucrose, D-glucose, D-(+)-raffinose, and the relatively lower citric acid and malic acid might contribute to the sweet taste of A. By screening the differentially expressed genes (DEGs) and correlation analysis of the DEGs and differentially accumulated metabolites, we deduced that the B cultivar might promote the conversion of glucose and fructose into intermediate compounds for downstream processes such as glycolysis. The tricarboxylic acid (TCA) cycle might also be enhanced compared to A, thus resulting in the differential accumulation of soluble sugars and organic acids, ultimately causing the taste difference between the two oriental melon cultivars. Our finding provides important information for further exploring the metabolic mechanisms of soluble sugars and organic acids in oriental melon.

## Introduction

Oriental melon (*Cucumis melo* var. *acidulus*), of the Cucurbitaceae family, is a sweet and aromatic melon that is highly popular worldwide, representing a large share of the produce market (Garcia-Mas et al., 2012; Lian et al., 2021). As one of the most economically important fruit crops, the fruits of oriental melon are rich in vitamins, minerals, and other health-promoting substances (Yano et al., 2020). The fruit is the main carbohydrate sink and depends on carbohydrate transport from the leaf source (Sonnewald and Fernie, 2018). Owing to the lack of stored starch reserves, oriental melon fruit requires a concurrent supply of photoassimilates from the leaves for sugar accumulation during the development process (Hubbard et al., 1990; Dai et al., 2006). Thus, the oriental melon is an attractive model for studying the dynamic changes of photoassimilates.

Sweetness is an important trait in the organoleptic quality assessment of melon, determined by the metabolite composition of the fruit, for example, sugars and organic acids (Vallone et al., 2013). In sweet melon, sucrose is the primary component determining fruit quality (Yamaguchi et al., 1977; Burger and Schaffer, 2007). Sucrose accumulation is a developmentally regulated process in the melon fruit mesocarp, which undergoes a metabolic transition from the early fruit growth stage to that of sucrose accumulation, covering a dozen enzymatic reactions (Dai et al., 2011). Additionally, the ratio of sugars to organic acids has a significant influence on the fruit quality (Umer et al., 2020). Generally, organic acid metabolism in the fruit is a complex physiological process, and the organic acid content is determined by the balance of acid synthesis and degradation (Tang et al., 2010). To date, various studies on sucrose and organic acid accumulation in melon have been conducted using transcriptome sequencing, genomic tools, and functional analysis, most of which have focused on only a few enzyme activities (Obando-Ulloa et al., 2009; Dai et al., 2011; Zhang et al., 2016). Thus, a systematic study of the metabolism and molecular mechanisms related to sugars and organic acids will prove to be informative.

In recent years, integrative analyses based on multifunctional “omics” approaches have offered an efficient means of identifying gene networks and their regulatory mechanisms in living systems (Fu et al., 2021; Wang et al., 2021). In particular, the combined analysis of the transcriptome and metabolome has been widely used for identifying the signaling pathways and mechanisms controlling sugar and organic acid accumulation in plant fruits. For example, Li et al. (2020) used temporal transcriptome analysis combined with targeted metabolomics to explore the mechanisms of differential sugar accumulation in two mango cultivars and found that the synthesis of sucrose and D-glucose, accompanied by the degradation of starch, directly contributed to high sugar accumulation in the fruit. Similar approaches have been employed in watermelon (Umer et al., 2020), kiwifruit (Xiong et al., 2020), apple (Xu et al., 2020), etc. However, such comprehensive studies elucidating the key gene networks controlling sugar and organic acid regulation in oriental melon fruit are lacking. Comparative analysis of contrasting cultivars offers a promising approach for identifying important traits controlling fruit quality.

In the present study, comprehensive transcriptome profiling and metabolite analysis were performed wherein the fruits of the oriental melon cultivars ‘Tianbao’ (2020B55, A), with high sugar content, and ‘Xiaocuigua’ (2020B23, B), with low sugar content, were compared at different developmental stages. Our study may help to elucidate the dynamics and differential expression of genes linked to the metabolism of sugars and organic acids in oriental melon.

## Results

### Total Soluble Solids and Total Acids of Different Ripening Fruits From Two Oriental Melon Cultivars at Different Ripening Stages

**FIGURE 1:**
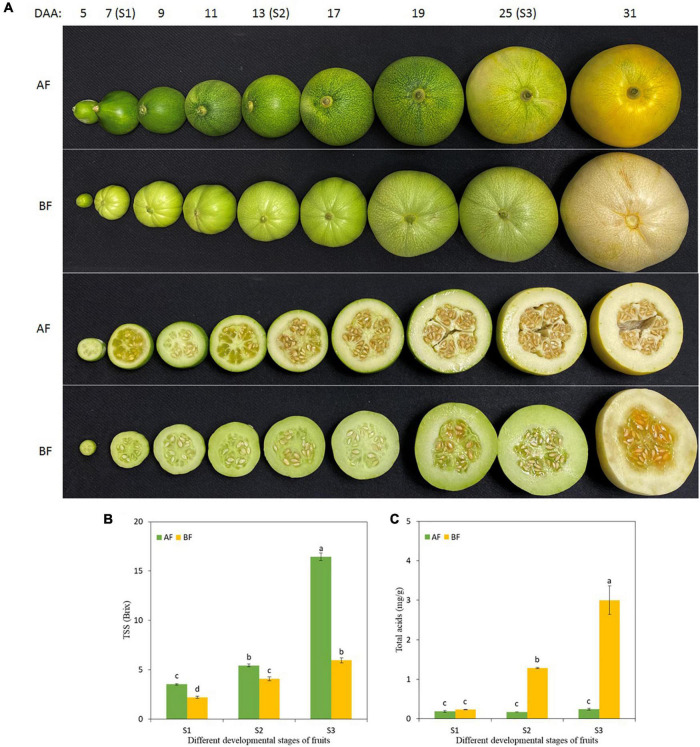
Changes in oriental melon fruits during consecutive ripening stages. **(A)** Pictures of oriental melon cultivars A and B fruits at different developmental stages, and the collection stages of fruit samples were S1 (7 DAA), S2 (13 DAA), and S3 (25 DAA). **(B)** TSS in the fruit during the three ripening stages. **(C)** Total acids in the fruit during the three ripening stages. Values are means ± standard errors (SE) (*n* = 3) and bars indicate SE. Significant differences between columns are indicated by different letters (Duncan’s test, *p* < 0.05).

The fruits of the two oriental melon cultivars were harvested at three ripening stages to investigate the changes in total soluble solids (TSS) and total acids in the fruits (Figure 1A). With the fruit development, the TSS of the A and B cultivar accumulated consistently and peaked at S3 [S1, the first stage of 7 days after anthesis (DAA); S2, 13 DAA; and S3, 25 DAA], but the level of TSS in each stage of A was significantly higher than in B (Figure 1B). Furthermore, there was no significant difference for total acids in the different developmental stages of the A fruits, while the total acids increased evidently with B fruit ripening (Figure 1C). The differences in TSS and total acids explained the differences between the natural sweetness of these two cultivars.

### Metabolite Analysis and Differential Accumulation of Metabolites

For the metabolite analysis, a principal component analysis (PCA) of the metabolite profiles of the 36 samples (6 different collecting groups × 6 replicates) was performed, which showed that the six groups could be separated in the PC1 × PC2 score plots (Supplementary Figure 1). A total of 4,346 metabolites were detected (Supplementary Table 2). Based on the screening criteria of variable importance in the projection (VIP) > 1 and *p* < 0.05 from all detected metabolites, a total of 961 differentially accumulated metabolites (DAMs) were investigated among the different comparison groups (Table 1). In the same ripening stage of A fruit (AF) vs. B fruit (BF), the DAMs of S3 were found to be more abundant than S1 and S2, implying that the main change in DAMs occurred in S3.

**TABLE 1:** Comparison of differential accumulated metabolites in the fruits of two oriental melon cultivars A and B.

Comparison groups	DAMs number	Up-regulated DAMs number	Down-regulated DAMs number
AFS1 vs. BFS1	285	133	152
AFS2 vs. BFS2	266	165	101
AFS3 vs. BFS3	413	228	185
AFS2 vs. AFS1	410	223	187
AFS3 vs. AFS1	420	239	181
AFS3 vs. AFS2	402	209	193
BFS2 vs. BFS1	382	179	203
BFS3 vs. BFS1	491	249	242
BFS3 vs. BFS2	305	168	137

Subsequently, the Kyoto Encyclopedia of Genes and Genomes (KEGG) enrichment analysis was used to further understand the biological mechanisms associated with the two oriental melon cultivars. Based on the statistical significance criterion for *p*-value less than 0.05, a total of 10–22 KEGG pathways were significantly enriched in different comparison groups in the fruits (Supplementary Table 3). Of these, metabolites involved in the metabolism of sucrose and organic acids were more differentially accumulated in the fruits, including ‘galactose metabolism,’ ‘pentose phosphate pathway,’ ‘fructose and mannose metabolism,’ ‘linoleic acid metabolism,’ and ‘arachidonic acid metabolism.’

**FIGURE 2:**
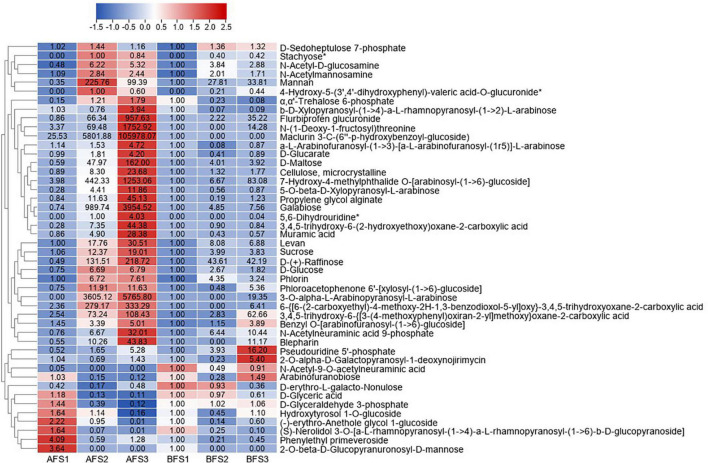
Heatmap of the DAMs of sugar metabolites during three fruit developmental stages of the oriental melon cultivars A and B. The three “A” fruit development stages are AFS1, AFS2, and AFS3, and three “B” fruit development stages are BFS1, BFS2, and BFS3. The DAMs were selected based on a FC value ≥ 2 and FC value ≤ 0.5 in any comparison groups of AF and BF. The FC values are displayed in the heatmap, which used the data of BFS1 as the calibrator, while the metabolites with asterisks used the data of AFS2 as the control (due to no detection in BFS1).

The differentially abundant sugar metabolites with fold-change (FC) values ≥ 2 and FC ≤ 0.5 in any of the comparison groups in the fruits were further selected, and the change patterns of which were displayed in a heatmap (Figure 2). As expected, the majority of sugar metabolites showed increasing trends from AFS2 and peaked at AFS3, and they accumulated more greatly in AFS3 compared to BFS3, for example, D-maltose, galabiose, sucrose, and D-(+)-raffinose. Maclurin 3-C-(6′′-p-hydroxybenzoyl-glucoside) increased evidently in A fruit ripening but was not detected in BFS2 and BFS3. The results imply that more abundant sugar metabolites might be crucial for the sweetness of A fruits. Owing to the screening criteria, the changes in D-fructose and fructose 6-phosphate were not included in Figure 2, and thus we analyzed their accumulation separately. We found that D-fructose increased gradually in S2 and then decreased in S3 in both A and B, the value of which in BFS3 was 2.0-fold higher than in AFS3. Fructose 6-phosphate decreased consistently both in A and B fruits.

**FIGURE 3:**
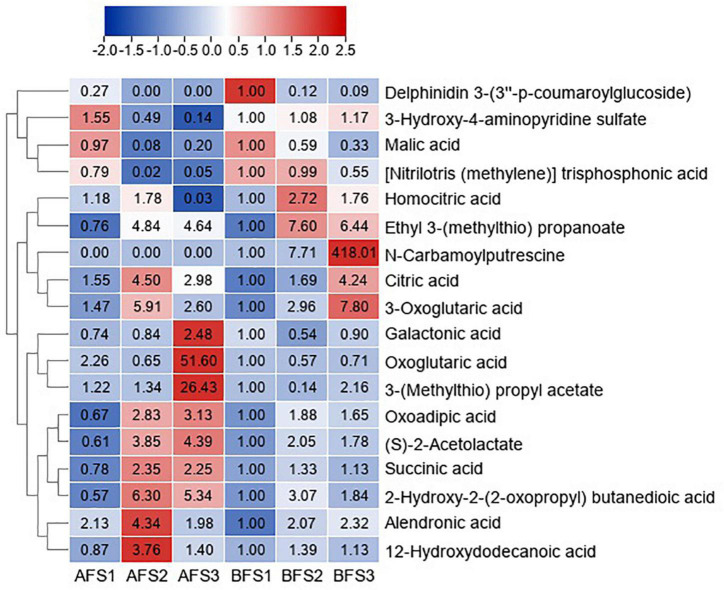
Heatmap of the DAMs of organic acids during three fruit developmental stages of oriental melon cultivars A and B. The three “A” fruit development stages are AFS1, AFS2, and AFS3, and three “B” fruit development stages are BFS1, BFS2, and BFS3, respectively. DAMs are selected based on VIP > 1 and *p* < 0.05 in any of the comparison groups in the fruits. The FC values are displayed in the heatmap, which used the data of BFS1 as the calibrator.

The DAMs of organic acids with VIP > 1 and *p* < 0.05 in any of the comparison groups in the fruits were also assessed, and the change patterns were displayed in a heatmap (Figure 3). The accumulation of malic acid continuously decreased with fruit ripening in both A and B. The level of citric acid increased and peaked in AFS2 and BFS3. N-carbamoylputrescine was not detected in any stages of AF, but it accumulated significantly in BFS3. Abundant organic acids accumulated to high levels at stages S2 and S3.

### Transcriptome Analysis and Differentially Expressed Genes

To identify differentially expressed genes (DEGs) in the fruits at different developmental stages, a total of 18 libraries from six groups were developed using transcriptomic analysis. The PCA showed that the 18 samples could be clearly assigned to six groups (Supplementary Figure 2). Overall, 123.13 G of clean data were mapped to the melon reference genome, and the match rate was in the range of 96.48–98.22%.

Using the threshold of *p* < 0.05 and FC value > 2, a total of 14,703 DEGs were identified among the six groups (Table 2). In all the comparison groups, the most DEGs accumulated in BFS3 vs. BFS1. The common and unique DEGs from the pairwise comparisons among the developmental stages are shown in Supplementary Figure 3. A total of 1,113 DEGs were uniquely expressed in BFS3 vs. BFS1, suggesting that a considerable proportion of the transcriptomic changes in the fruits of oriental melon cultivar B occurred during the maturation process.

**TABLE 2:** DEGs statistics in the fruits of two oriental melon cultivars.

Comparison groups	Total DEGs	Down-regulated DEGs	Up-regulated DEGs
AFS1 vs. BFS1	1,185	618	567
AFS2 vs. BFS2	5,077	3,381	1,696
AFS3 vs. BFS3	5,737	2,860	2,877
AFS2 vs. AFS1	7,797	4,594	3,203
AFS3 vs. AFS1	8,974	5,513	3,461
BFS2 vs. BFS1	5,578	3,206	2,372
BFS3 vs. BFS1	10,667	6,233	4,434

The KEGG analysis of the DEGs among different comparison groups provided additional information about the enriched biological pathways (Supplementary Table 4). Based on the statistical significance criterion for multiple testing correction (adjusted *p*-value), ‘carbon metabolism’, ‘carbon fixation in photosynthetic organisms’, ‘plant hormone signal transduction’, and ‘photosynthesis-antenna proteins’ were significantly enriched (*q*-value ≤ 0.001) throughout leaf and fruit development. Additionally, ‘polyketide sugar unit biosynthesis’ was extremely enriched (*q*-value ≤ 0.001) in AFS2 vs. BFS2, AFS3 vs. BFS3, and AFS3 vs. AFS1. ‘Starch and sucrose metabolism’, and ‘amino sugar and nucleotide sugar metabolism’ (*q*-value ≤ 0.001) were evidently enriched in BFS2 vs. BFS1.

### Combined Metabolomic and Transcriptomic Analyses in the Fruits

**FIGURE 4:**
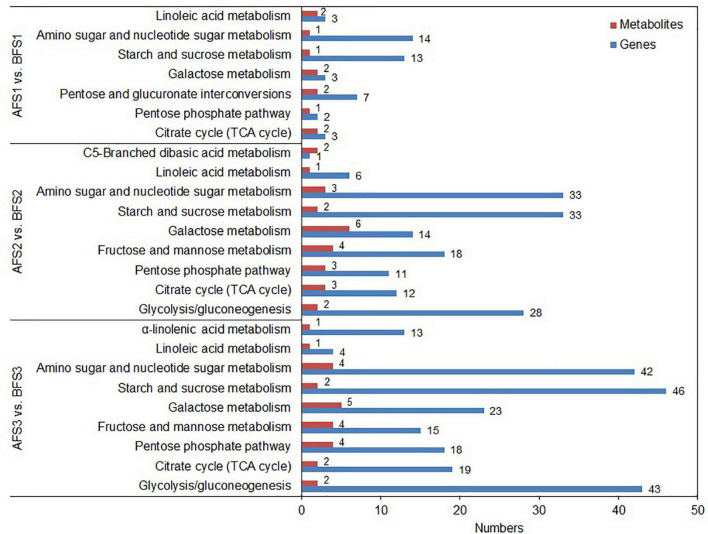
Integrated analyses of the transcriptome and metabolome of the KEGG pathways related to sugars and organic acids. Three comparison groups of AFS1 vs. BFS1, AFS2 vs. BFS2, and AFS3 vs. BFS3 are selected. The specific name of the KEGG pathway is provided on the left of the bar chart, and the numbers of relative DEGs and DAMs are on the right of the bar.

To further explore the relationship between gene expression and metabolite accumulation in oriental melon fruits, correlation tests and conjoint biological annotations were conducted for the comparison of AF vs. BF. By combining the metabolomics and transcriptomics data, we identified the common altered KEGG pathways relative to sugar and organic acid metabolism and identified the DEGs and DAMs between A and B fruits (Figure 4 and Supplementary Table 5). In all three comparison groups (AFS1 vs. BFS1, AFS2 vs. BFS2, and AFS3 vs. BFS3), ‘starch and sucrose metabolism’ and ‘amino sugar and nucleotide sugar metabolism’ included more DEGs, while ‘pentose and glucoronate interconversions’ (AFS1 vs. BFS1) and ‘glycolysis/gluconeogenesis’ (AFS2 vs. BFS2 and AFS3 vs. BFS3) were the third most enriched pathways. A total of 10, 20, and 19 DAMs related to sugar and organic acid metabolism were found in the common KEGG pathway analysis.

**FIGURE 5:**
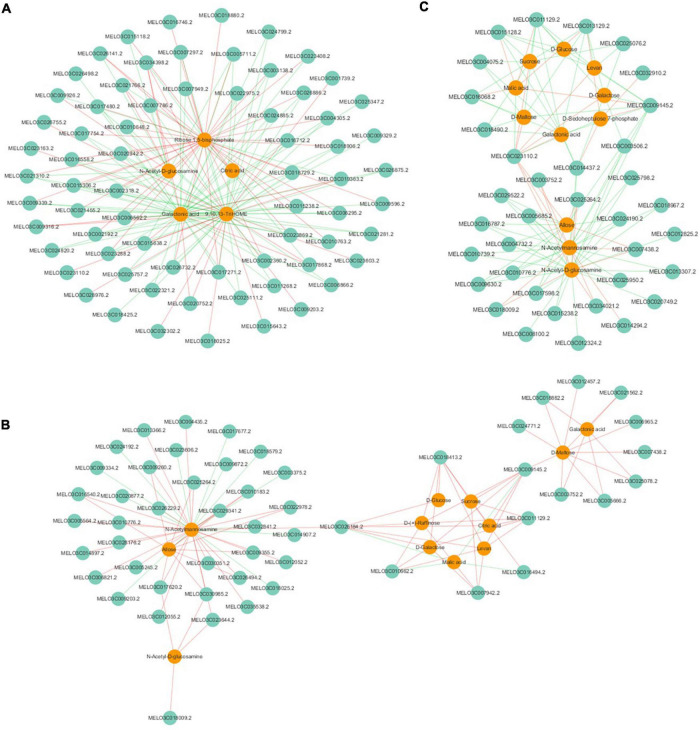
Correlation network of DEGs and DAMs involved in sugar and organic acid metabolism. **(A)** AFS1 vs. BFS1, **(B)** AFS2 vs. BFS2, **(C)** AFS3 vs. BFS3. Green circles indicate genes, and orange circles indicate metabolites. Lines colored in “red” and “green” represent positive and negative correlations, respectively, as determined by a Pearson’s correlation coefficient > 0.8 or < −0.8 (*q*-value < 0.1), respectively.

Pearson’s algorithm was used to calculate the correlations between the top-100 DEGs and DAMs. From the response intensity data of the correlation analysis, network analyses of DEGs and DAMs related to sugar and organic acid metabolism were found and intuitively illustrated both in AFS2 vs. BFS2 and AFS3 vs. BFS3 (Figure 5). The DAMs in AFS2 vs. BFS2, such as sucrose, D-glucose, D-galactose, D-(+)-raffinose, citric acid, and malic acid, were regulated positively or negatively by the DEGs, such as the genes encoding NADP-dependent malic enzyme (NADP-ME, MELO3C011129.2), pyruvate decarboxylase 1 (PDC1, MELO3C009145.2), and aconitate hydratase 1 (ACO1, MELO3C007942.2). A gene encoding sugar will eventually be exported transporters (SWEET10, MELO3C026184.2) was negatively correlated with malic acid (*r* = −0.99) and citric acid (*r* = 0.95), and positively correlated with D-galactose (*r* = 0.96), D-glucose (*r* = 0.87) and D-(+)-raffinose (*r* = 0.82). The accumulation of sucrose in AFS3 vs. BFS3 was regulated negatively by the genes encoding isocitrate dehydrogenase (IDH, MELO3C025076.2), and ACO1-1 (MELO3C014437.2), while the content of malic acid in AFS3 vs. BFS3 was regulated positively by the genes encoding NADP-ME (MELO3C011129.2), PDC1 (MELO3C009145.2), ACO1-1 (MELO3C014437.2), and IDH (MELO3C025076.2). The above DEGs and DAMs might be important contributors to the differences between the two oriental melon cultivars A and B.

### Candidate Genes Related to the Different Soluble Sugar Metabolisms of the Two Melon Cultivars

**FIGURE 6:**
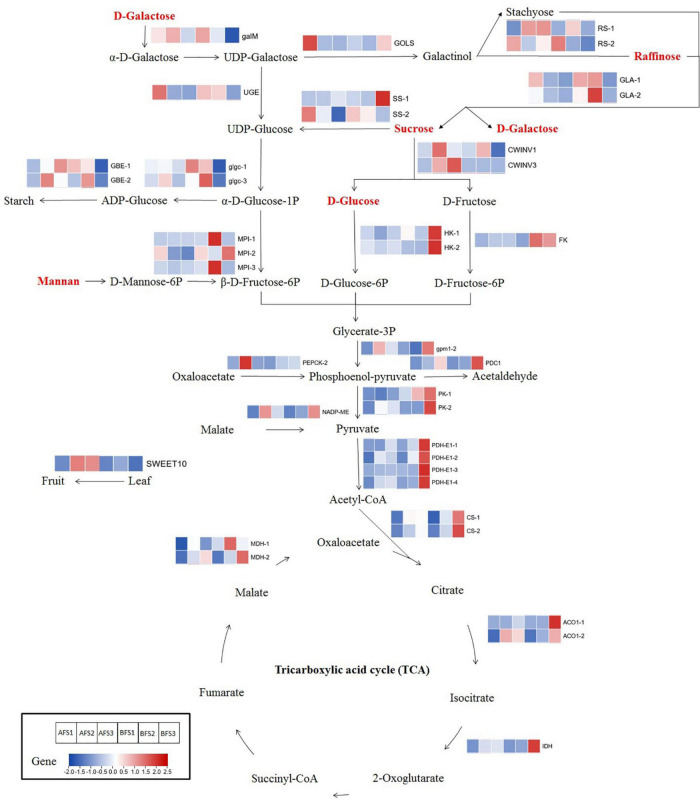
The different soluble sugar and organic acid biosynthetic pathways in the two oriental melon cultivars fruits. Enzyme names are next to their expression patterns at different ripening stages in the oriental melon fruits. The expression pattern is reflected by a fold-change value, which used the data of BFS1 as the control. The metabolites in red represent highly accumulating metabolites in the mature fruits of AFS3 compared to BFS3. GalM, aldose 1-epimerase; GOLS, inositol 3-alpha-galactosyltransferase; RS, raffinose synthase; SS, sucrose synthase; UGE, UDP-glucose 4-epimerase; GBE, 1,4-alpha-glucan branching enzyme; glgc, glucose-1-phosphate adenylyltransferase; INV, invertase; GLA, alpha-galactosidase; MPI, mannose-6-phosphate isomerase; HK, hexokinase; FK, fructokinase; gpm1, phosphoglycerate mutase; PDC1, pyruvate decarboxylase 1; PK, pyruvate kinase; PDH-E1, pyruvate dehydrogenase E1 component; CS, citrate synthase; ACO1, aconitate hydratase 1; IDH, isocitrate dehydrogenase; MDH, malate dehydrogenase; NADP-ME, NADP-dependent malic enzyme; PEPCK, phosphoenolpyruvate carboxykinase; SWEET10, sugar will eventually be exported transporters.

Some important genes associated with soluble sugar metabolism were found to be differentially expressed and might have considerable effects on the varied sweetness of the two oriental melon cultivars at different ripening stages of fruit development (Figure 6 and Supplementary Table 6). An *aldose 1-epimerase* (*galM*) gene showed higher expression in AFS3 than in BFS3 (31.9-fold higher). An *inositol 3-alpha-galactosyltransferase* (*GOLS*) gene exhibited a continuous decline in AF but first decreased and then increased in expression in BF, and the FC value in BFS3 was 17.3 times higher than in AFS3. Two *raffinose synthase* (*RS*) genes and two *invertase* (*INV*) genes showed relative higher expression in various AF stages compared to BF. Two *sucrose synthase* (*SS*) genes, *hexokinase* (*HK*) genes, *UDP-glucose 4-epimerase* (*UGE*) gene, and *fructokinase* (*FK*) gene were found to be upregulated in BF compared to AF. Furthermore, two *glucose-1-phosphate adenylyltransferase* (*glgc*) genes and two *1,4-alpha-glucan branching enzyme* (*GBE*) genes showed higher expression in AFS3 compared to BFS3. By contrast, two *trehalose 6-phosphate phosphatase* (*TPS*) genes, two *alpha-galactosidase* (*GLA*) genes, and three *mannose-6-phosphate isomerase* (*MPI*) genes showed higher expression in BFS2 or BFS3 than at the same stage in AF. In addition, the expression of *SWEET10* showed that it was strongly and significantly upregulated in AFS2 (3.8-fold higher than BFS2) and AFS3 (17.5-fold higher than BFS3), implying that SWEET10 played a major role in sugar transport and accumulation in the A cultivar.

### Candidate Genes Linked to Different Organic Acid Metabolisms of the Two Melon Cultivars

Combined DEG identification, correlation analysis, and the common KEGG pathway analysis of the DEGs and DAMs related to organic acid metabolism indicated that some candidate genes linked to the metabolism of organic acids showed significantly different expressions in the two melon cultivars (Figure 6). Among them, the majority of genes in AF showed increasing trends and peaked in AFS2 [such as *NADP-ME* and *phosphoenolpyruvate carboxykinase* (*PEPCK-2*) gene] or AFS3 (such as *ACO1-1* and *PDC1*). By contrast, most genes in BF were upregulated and peaked in BFS3; for example, *ACO1-1* (1564.7-fold higher in BFS3 than BFS1), *PDC1*, and *NADP-ME*. Interestingly, all these genes were much more abundant in BFS3 compared to BFS3, varying from 1.4- to 6.2-fold.

### Other Candidate Genes Manipulate Different Ripening Behaviors of the Two Melon Cultivars

To analyze other factors manipulating the ripening processes of the two melon cultivars, some crucial genes related to ethylene signal transduction were found differentially expressed (Table 3). The expression of an *ethylene response sensor 1* (*ERS1*) gene was gradually decreased with AF ripening, while the expression of which was increased firstly then decreased with BF ripening and peaked at BFS2. Two *ethylene-responsive transcription factors* (*ERFs*) were more highly expressed in AFS1 than BFS1 but were highly expressed in BFS2 and BFS3. Besides, a *mitogen-activated protein kinase 6* (*MPK6*) homology gene was repressed with fruit ripening both in A and B cultivars, however, the expression of it was much higher in BFS2-BFS3 than AFS2-AFS3.

**TABLE 3:** DEGs information related to ethylene signal transduction and carotenoid metabolism.

Gene ID	Gene name	FPKM value					
		AFS1	AFS2	AFS3	BFS1	BFS2	BFS3
MELO3C015961.2	*ERS*	68.19	20.48	15.45	60.86	100.90	39.55
MELO3C022985.2	*ERF1B*	15.86	0.13	0.31	7.11	5.27	0.06
MELO3C006430.2	*ERF1B*	47.46	2.06	3.71	26.05	36.40	21.31
MELO3C011444.2	*MPK*	51.15	21.21	7.35	55.05	47.86	12.22
MELO3C016185.2	*PSY*	25.54	4.06	1.12	18.03	23.63	0.78
MELO3C017772.2	*PDS*	24.05	235.76	121.06	28.71	86.96	370.77
MELO3C024674.2	*ZDS*	28.69	91.27	59.20	32.10	35.32	60.64
MELO3C016224.2	*CCD*	28.94	143.98	462.05	28.87	49.16	562.54
MELO3C022291.2	*CCD*	0.19	73.16	60.81	0.05	0.24	91.58

To investigate the pathway of carotenoid biosynthesis, some carotenoid metabolism-related genes were identified and significantly differentially expressed between AF and BF (Table 3). *Phytoene synthase (PSY)* gene was continuously decreased with AF ripening, which was increased and peaked at BFS2 then decreased, and the expression level of which was 5.8 times in BFS2 compared to AFS2. The trends of *phytoene desaturase (PDS)* and *ζ-carotene desaturase (ZDS)* genes were similar, increasing firstly then decreasing with AF ripening, but consistently increasing with BF ripening. Besides, *carotenoid cleavage dioxygenase (CCD)* genes increased drastically with fruit ripening both in A and B, the expression levels of *CCD-1* in S3 were 16.0–19.5-fold higher than the levels in S1, the levels of *CCD-2* in S3 were 320.1–1831.6-fold higher than the levels in S1. The levels of *PDS, ZDS, and CCDs* were much higher in AFS2 than in BFS2, while the levels of *PDS and CCDs* were significantly lower in AFS3 than in BFS3.

### Validation of Candidate DEGs by Quantitative Real-Time PCR

**FIGURE 7:**
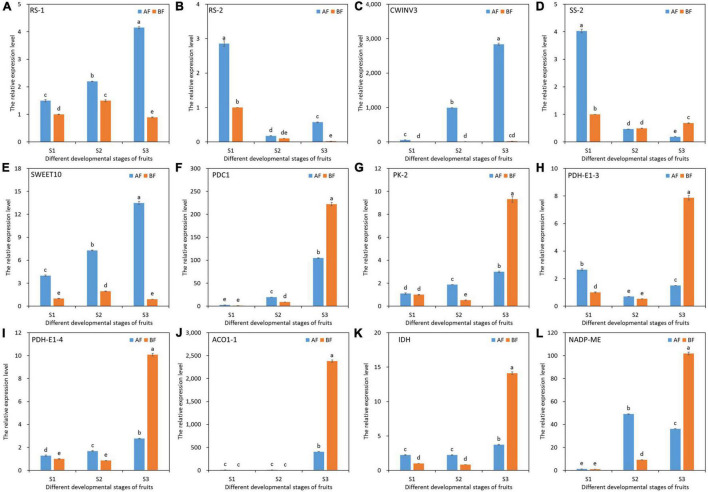
qRT-PCR validation of 12 selected DEGs related to soluble sugar and organic acid metabolism. The enzymes names are labeled on the upper left of the separated figures. *GAPDH* (CuGenDB number: MELO3C008219) was used as the reference gene. The data of BFS1 were used as the control to calculate the relative expression levels. **(A)** RS-1. **(B)** RS-2. **(C)** CWINV3. **(D)** SS-2. **(E)** SWEET10. **(F)** PDC1. **(G)** PK-2. **(H)** PDH-E1-3. **(I)** PDH-E1-4. **(J)** ACO1-1. **(K)** IDH. **(L)** NADP-ME.

To validate the relative expression patterns of the unigenes from our RNA-seq analysis, we selected 12 key DEGs related to soluble sugar and organic acid metabolism and performed quantitative real-time PCR (qRT-PCR) expression analysis between the two oriental melon cultivar fruits at different developmental stages. The expression patterns of all selected genes from the qRT-PCR were similar to the RNA-Seq, with an average *r-*value of 0.9 (the *r*-value varied from 0.73 to 1.00), confirming the reliability of the RNA-seq data and subsequent analyses (Figure 7).

## Discussion

Sugar content and organic acid accumulation are important criteria for oriental melon fruit quality and taste. Therefore, it is of great importance to reveal the molecular mechanisms involved in sugar accumulation and organic acid metabolism in oriental melon fruit. The combination of different omics techniques can provide a more in-depth understanding of the mechanisms of sugar accumulation and organic acid metabolism in ripening fruits, such as loquat (Zou et al., 2020), watermelon (Umer et al., 2020), mango (Li et al., 2020), and pummelo (Zhu et al., 2020). In addition, comparative analysis between contrasting cultivars is an efficient approach for identifying differential pathways, expressed genes, or metabolite variations that control important traits (Wang et al., 2019; Fei et al., 2021; Xiao et al., 2021). Compared with the oriental melon cultivar B, a total of 961 DAMs were detected in the fruits at different ripening stages in this study, while a total of 14,703 DEGs were also found. Consequently, the abundant metabolites and DEGs identified in this study will not only provide substantial information for the high-quality genetic improvement of oriental melon but also offer a valuable reference for other crops of the Cucurbitaceae family.

### Accumulation Changes of Soluble Sugars and Organic Acids in the Oriental Melon Fruits

In sweet melon, the accumulation of sucrose, glucose, and fructose is the main factor determining fruit quality (Vallone et al., 2013). Sucrose accounts for nearly all the variation in total sugar content in both low- and high-sugar accumulating accessions (Argyris et al., 2015). In the present study, the sucrose and D-glucose content increased evidently and peaked at AFS3, and both sucrose and D-glucose increased quickly in BFS2 and decreased in BFS3. Compared to these two sugars, the levels of D-fructose did not vary significantly throughout the ripening process for A and B. The rapid accumulation of sucrose and D-glucose in the medium and late ripening stages of the A cultivar might determine the different sweetness compared to the B cultivar. A similar accumulation pattern of sucrose has been observed in other sweet melon cultivars (Zhang and Li, 2005; Burger and Schaffer, 2007; Zhang et al., 2016). Furthermore, the catabolism of the photosynthates raffinose oligosaccharides (raffinose and stachyose) contributes to the sustained accumulation of sucrose in ripe fruits (Dai et al., 2011; Ren et al., 2021). From our comparison between A and B, D-(+)-raffinose varied widely, while stachyose exhibited limited change. The content of D-(+)-raffinose was much higher in AFS2-S3 than in BFS2-S3, indicating that a greater degree of hydrolysis occurred in the B fruits. Our result was not consistent with the research of Ren et al. (2021). We propose that the hydrolysis of D-(+)-raffinose is not the crucial factor contributing to the differences in sucrose and glucose accumulation patterns in the current study, but rather that the subsequent resynthesis process of sucrose might be the possible reason. This speculation needs to be further explored.

Organic acids play a crucial role in fruit nutrition, and their contents are determined by the balance between acid synthesis and degradation (Zampini et al., 2008). Moderate concentrations of organic acids can enhance the taste of the fruit, but a high acid content often reduces the quality of fruits (Gao et al., 2018). Citric acid and malic acid are the major organic acids in melon fruits (Flores et al., 2001). We detected both citric acid and malic acid in A and B fruits, and malic acid showed a continuous decline throughout fruit ripening in A and B, and no major difference was found when compared with A and B. On the contrary, citric acid increased first in AFS2 and then declined in AFS3, but its content increased consistently with B fruit ripening. This reflected the predominance of citric acid in B fruit development and might explain the difference in acids between A and B fruits. Similar change patterns of citric acid were observed in two melon cultivars with different levels of sweetness, namely ‘Xuelihong’ and ‘Flavor No. 3′ (Tang et al., 2010). In summary, the abundant and varied sugars and organic acids contributed to the different tastes of the two oriental melon cultivars A and B.

### Gene Regulation Varied Sugar Metabolism in the Two Oriental Melon Cultivar Fruits

Generally, sucrose is first produced *via* the photosynthesis of the leaves (source tissues) and is subsequently transported to and stored in the fruits (sink tissues) (Chen et al., 2015). This long-distance translocation of sucrose is controlled by sucrose transporters and SWEET efflux proteins, and SWEETs are functionally characterized as having substrate preferences for sucrose, glucose, or fructose (Chen et al., 2012; Shammai et al., 2018). Zhang et al. (2019) revealed that *VvSWEET10* overexpression in grapevine calli and tomatoes increased glucose, fructose, and total sugar levels significantly. In the present study, we noticed a high correlation between *SWEET10* expression and D-glucose accumulation, while *SWEET10* was strongly upregulated in A compared to B, suggesting that A might transport more sugars to the fruit compared with B, thereby resulting in a significant increase in sugar content. The specific function of SWEET10 will be further explored.

Beyond sucrose, raffinose family oligosaccharides (RFOs), represented by raffinose and stachyose, are ubiquitous in the fruits and are often used for the transport of carbon skeletons from source to sink tissues (Lahuta et al., 2014). The biosynthesis of RFOs is initiated by the synthesis of galactinol from UDP-galactose catalyzed by GOLS and is transferred to raffinose yield by RS, as well as stachyose yield by stachyose synthase (STS), following which the hydrolysis of raffinose and stachyose is released to sucrose and galactose by GLA (Egert et al., 2013; Lahuta et al., 2014). One *GOLS* gene in our paper showed a very high level in AFS1, and then declined quickly, but it increased significantly in BFS3, indicating that the enzyme encoded by this gene functioned differently in the ripening stages of A and B fruits. Two *RS* genes and *GLA* genes exhibited different expression trends; the *RS* genes exhibited higher expression in AFS2-S3 than BFS2-S3, whereas the *GLA* genes showed higher expression in BFS2-S3 compared to AFS2-S3, and these changes were consistent with the accumulation of raffinose in A and B fruits, respectively. It was speculated that, during the ripening of the fruit, more raffinose was accumulated but hydrolyzed less in A compared to B, as regulated by *RS* genes and *GLA* genes.

Once sucrose is transported into fruit cells, a complex metabolism will be engaged. Sucrose can be converted to fructose and glucose by INV, and SS can also catalyze sucrose to fructose and UDP-glucose (Koch, 2004). We detected higher expression of *SS-1* in BFS3 than AFS3 and evidently higher expression of *CWINV3* in AFS2-S3 than BFS2-S3. Our results implied that B prioritized the conversion of sucrose to UDP-glucose and fructose regulated by *SS-1*; however, A preferred the conversion of sucrose to glucose and was regulated by *CWINV3*. The resulting glucose and fructose are phosphorylated to glucose 6-phosphate (G6P) and fructose 6-phosphate (F6P) by HK and FK (Xiong et al., 2020). Compared to the AF ripening stages, two *HK* genes and one *FK* gene showed higher expression in the BF ripening stages, especially at S2 and S3, suggesting that the B cultivar promoted the expression of *HK* and *FK* genes that convert glucose and fructose into intermediate compounds for downstream processes such as glycolysis (Figure 6). Likewise, between the comparison of two contrasting cultivars (high-sweetness and low-sweetness), the expression of *HK* and *FK* was higher in the low-sweetness cultivar, and the high-sweetness cultivar repressed the expression of genes converting sugars into intermediate compounds (Li et al., 2020). Furthermore, MPI is a metal-dependent isomerase catalyzing D-mannose-6-phosphate (M6P) to F6P, using mannan as the substrate (Sigdel et al., 2015). *MPI* genes were found to have higher expression in BFS2 or BFS3 compared to the same stages of AF, reflecting that a greater portion of M6P was converted to F6P in BF, thus explaining the phenomenon of greater mannan accumulation in the fruits of A. Additionally, the *glgc* and *GBE* genes related to starch synthesis exhibited similar change patterns in the A and B ripening stages, but their FC values in AFS3 were much higher than in BFS3, indicating that A allowed greater starch synthesis in the mature fruits than B.

### Difference Between Pyruvate Metabolism and Tricarboxylic Acid Cycle in the Two Oriental Melon Cultivar Fruits

Pyruvate metabolism and the tricarboxylic acid (TCA) cycle are ubiquitous throughout nature, playing important roles in energy metabolism, gluconeogenesis, lipogenesis, and amino acid synthesis (De Meirleir et al., 2006). Phosphoenol-pyruvate is produced by the conversion of oxaloacetate through PEPCK and is then converted to pyruvate by pyruvate kinase (PK). The resulting pyruvate can be converted into acetyl coenzyme A (acetyl-CoA) by pyruvate dehydrogenase (PDH), followed by further oxidation in the TCA cycle (Patel and Roche, 1990). From our results, the expression of *PEPCK-2* was upregulated and peaked at AFS2; meanwhile, it increased consistently during B fruit ripening, indicating that the conversion of phosphoenol-pyruvate occurred earlier in A fruits compared to B fruits. *PK* and *PDH-E1* had similar expression patterns both in A and B in the last ripening stages; they exhibited significantly higher expression in BFS3 compared to AFS3, providing a clue that pyruvate metabolism might be more pronounced in B fruits than in the fruits of A.

In the TCA cycle, CS directly synthesizes citric acid by first catalyzing acetyl Co-A, following which citric acid is degraded to isocitric acid by ACO, and then isocitric acid is transported and produces 2-oxoglutarate by IDH (De Meirleir et al., 2006; Umer et al., 2020). In our study, the *CS*, *ACO*, and *IDH* genes all showed elevated expression during oriental melon fruit development, and their expression abundance was evidently higher in BF than in AF. The results indicated that citric acid metabolism was strengthened in B and was regulated by these genes. MDH and NADP-ME are enzymes linked to malic acid biosynthesis and degradation in the fruit (Sweetman et al., 2009). We identified two *MDH* genes and one *NADP-ME* gene with varied expression trends in the ripening processes of A and B, suggesting that these are key players in the metabolism of malic acid. *NADP-ME* showed extremely high expression in AFS2 and then decreased in AFS3, but showed elevated expression with B fruit ripening. It was interesting to note that the expression of *NADP-ME* was significantly correlated to malic acid content. Thus, we speculated that the degradation of malic acid occurred early in the A fruit ripening process, and this process proceeded continuously in B, thus resulting in the relatively higher accumulation of malic acid in BFS2-S3. In summary, compared to A, the TCA cycle was more greatly promoted in the fruit of B during ripening, influencing the organic acid contents and ultimately causing the taste differences between the two oriental melon cultivars.

### Other Factors Affecting Fruit Quality in the Two Oriental Melon Cultivars

Ethylene is essential for the ripening of climacteric fruits in plants by promoting the rapid change in sweetness, color, firmness, and aroma (Oren et al., 2022). The climacteric ripening of melon is characterized by a rise in respiration rate and increased ethylene levels (Sato-Nara et al., 1999). In the present study, we found the KEGG pathway “plant hormone signal transduction” of DEGs was top enriched in the comparison group of AFS2 vs. BFS2, and also some genes of the pathway for ethylene signal transduction were differentially expressed in AF and BF. The ESR gene encodes an ethylene receptor, is a negative regulator of ethylene signaling and functions the upstream of the pathway (Chen et al., 2005). Nukui et al. (2004) found that transgenic *Lotus japonicus* plants expressing the *Cm-ERS1/H70A* gene did not produce fruit by destroying the ethylene-binding ability of the plants. Moreno et al. (2008) further speculated that *ERS* is responsible for the QTLs of fruit firmness and climacteric ripening. In the present study, *ERS1* showed different expressions in the different developmental stages of AF and BF, implying that ERS1 might be involved in AF and BF ripening *via* transcriptional regulation patterns. Furthermore, ERF transcription factors are located downstream of the ethylene signal transduction pathway and are involved in ethylene response in plants (Xiao et al., 2013). While, across a diverse collection and a large diallele population, Oren et al. (2022) also found a candidate gene *ERF* within QTL genomic intervals (related to earliness and fruit ripening traits in melon). Thus, two differentially expressed *ERF*s from our results suggested that they might play important roles in ethylene—involved different fruit ripening for A and B. Besides, the expression of *MPK6* in AF and BF might indicate the involvement of a MAP-kinase-like signaling cascade in the regulation of ethylene signaling. The specific functions of the above candidate genes need to be further validated.

Carotenoid accumulation is also an important component of melon fruit quality (Feder et al., 2019). Carotenoid accumulation during melon fruit ripening is correlated well with the transcriptional levels of biosynthetic genes, including *PSY*, *PDS*, and *ZDS* (Harel-Beja et al., 2010). Chayut et al. (2015) observed that the upregulation of *PSY-1*, *PDS*, and *ZDS* with fruit ripening of melon inbred lines “Dulce” and “Tam-Dew” was accompanied by enhanced carotenoids levels. From our results, *PSY*, *PDS*, and *ZDS* all showed significant expression differences between AF and BF ripening processes, implying their key roles in regulating different carotenoid biosynthesis of A and B. However, the drastically downregulated expression of *PSY* in AF ripening might reduce carotenoid biosynthesis of AF. Thereafter, the higher expression of *PDS* and *ZDS* in AFS2 than in BFS2 and the lower level of *PDS* in AFS3 than in BFS3 might provide a clue that carotenoid biosynthesis has earlier occurred in AF than BF. Besides, the prominent upregulation of two *CCD* genes during fruit maturation in both A and B was similar to the observation of Ibdah et al. (2006). The opposite expression trends of two *CCD* genes in AFS2 vs. BFS2 and AFS3 vs. BFS3 further support our hypothesis for the earlier occurred carotenoid biosynthesis in AF than BF. However, we did not detect the related products of the carotenoid pathway through the present method, thus the relationships between these genes expression trends and carotenoid accumulation were unknown.

## Materials and Methods

### Plant Material and Growth Conditions

Two oriental melon cultivars with different sweetness and acidities were used as the experimental materials. The high-sweetness oriental melon cultivar ‘Tianbao’ (A, 2020B55) is a currently widely cultivated commercial cultivar in China, while ‘Xiaocuigua’ (B, 2020B23) is a low-sweetness oriental melon cultivar. The plant materials were planted in the greenhouse with the day/night temperature of 23–28°^°^C/18–22°C at the melon breeding base in Gaolan county (36°16′49′′, 103°37′57′′), China, on March 15, 2020. The base belongs to the Vegetable Research Institute, Gansu Academy of Agricultural Sciences. The plants were grown with a 45-cm distance between seedlings in each row and a 50 cm distance between rows and were under the same local management practices (soil management, irrigation, fertilization, and disease control).

Fruit samples were collected at three different developmental stages (S1, S2, and S3), which were marked as AFS1, AFS2, AFS3, and BFS1, BFS2, BFS3, respectively (Figure 1A). The flesh was obtained from the center-equatorial portion of each fruit after removing the pericarp. Then the samples were then immediately transferred to liquid nitrogen and stored at −80°C for the following analyses. At each time point, three fruits were harvested from three different plants and used as a replicate, and three replicates were used for TSS, total acid measurement, and transcriptome sequencing, while six replicates were used for metabolomics analysis.

### Total Soluble Solids and Total Acid Measurement

After homogenizing the collected oriental melon fruit samples, TSS (Brix%) and total acids were determined in a digital refractometer ATAGO PAL-BX/ACID F5 (ATAGO Co., Ltd., Tokyo, Japan) at room temperature (Villegas et al., 2009).

### RNA Isolation and Transcriptome Sequencing

Total RNA was extracted using the Plant RNA Kit (50) (Omega) following the manufacturer’s protocol. The RNA integrity was evaluated using an Agilent 2100 Bioanalyzer (Agilent Technologies, Santa Clara, CA, United States) (Duan et al., 2020). The samples with RNA Integrity Number (RIN) ≥ 7 were subjected to the subsequent analysis. The libraries were constructed using a TruSeq Stranded mRNA LTSample Prep Kit (Illumina, San Diego, CA, United States) according to the manufacturer’s instructions. Then these libraries were sequenced on the Illumina NovaSeq 6000 platform from OE Biotech Co., Ltd. (Shanghai, China) and 150 bp paired-end reads were generated. Raw reads were first processed using Trimmomatic for quality control to obtain clean reads (Bolger et al., 2014). The clean reads were mapped to the melon genome (ERP001463) (Garcia-Mas et al., 2012) using HISAT2 (Kim et al., 2015).

Gene expression levels were reflected by fragments per kilobase per transcript per million mapped reads (FPKM) (Roberts et al., 2011). The FPKM value of each gene was calculated using Cufflinks, and the read counts of each gene were obtained by HTSeqcount. Differential expression analysis was performed using the DESeq (2012) R package (Anders and Huber, 2013). The DEGs were confirmed using the threshold of *p* < 0.05 and FC value > 2. Hierarchical cluster analysis using the Short Time-series Expression Miner (STEM) program (version 1.3.11) was performed to demonstrate the expression pattern of genes in different groups and samples. Gene Ontology (GO) enrichment and KEGG pathway enrichment analysis of DEGs were performed using R based on the hypergeometric distribution.

### Metabolite Detection and Data Analysis

A total of 80 mg of sample, 1 ml of a mixture of methanol and water (7/3, vol/vol), and 20 μl internal standard of 2-chloro-L-phenylalanine (0.3 mg/ml) dissolved in methanol were mixed together in a 1.5 ml Eppendorf tube and then ground at 60 Hz for 2 min, ultrasonicated at room temperature for 30 min after vortexing, and then placed at −20°C overnight. After centrifugation at 13,000 rpm at 4°C for 15 min, 150-μl supernatants were filtered using 0.22 μm microporous membrane prior to analysis on a Shimadzu Nexera UPLC system (Kyoto, Japan) and a Q-Exactive-HF mass spectrum detector (Thermo Fisher Scientific, Waltham, United States).

The acquired LC-MS raw data were analyzed using Progqenesis QI software (Waters Corporation, Milford, United States) based on the public databases Human Metabolome Database (HMDB), Lipidmaps (v2.3), and METLIN and a self-built database (Luming Biotech CO., Ltd., Shanghai, China). Quality-control (QC) samples were prepared by mixing all samples to determine the reproducibility of the obtained results (Jiang et al., 2021). PCA and orthogonal partial least-squares-discriminant analysis (OPLS-DA) was conducted to visualize the metabolic alterations among the experimental groups (Jia et al., 2019). The metabolites were considered reliable when their scores were higher than 36 (the full score is 60). The metabolites with VIP > 1 and *p* < 0.05 were considered as DAMs. The metabolite pathways were searched on the non-commercial KEGG database.

### Quantitative Real-Time PCR Validation

A total of 12 genes related to soluble sugar and organic acid metabolism were selected for analysis using qRT-PCR. The cDNA was synthesized from total RNA using the PrimeScript*™* RT reagent Kit with gDNA Eraser (Perfect Real Time) (TaKaRa, Dalian, China) according to the manufacturer’s instructions. *GAPDH* (CuGenDB number: MELO3C008219)^1^ was used as an internal control gene and the qRT-PCR method was based on Duan et al. (2020). The qRT-PCR analysis of each sample was performed in triplicate. The primers were designed with Primer Premier 5.0 software and synthesized by (OE biotech Co., Ltd., Shanghai, China) (Supplementary Table 1).

### Statistical Analyses

All the data are presented as means ± standard errors (SEs). ANOVA was performed using SPSS statistical software (Version 16.0, SPSS Inc., Chicago, IL, United States). Duncan’s multiple comparisons were used to detect differences among means at a significance level of *p* < 0.05. Pearson’s correlation analysis (two-tailed) was performed to compare the FPKM values from the RNA-seq and qRT-PCR expression profiles.

## Conclusion

In the present study, integrated analysis of metabolic and transcriptomic analyses was performed to investigate the gene networks controlling the metabolism of sugars and organic acids in the two oriental melon cultivars ‘Tianbao’ and ‘Xiaocuigua’. The key metabolites and candidate genes related to sugar and organic acid metabolism were screened, and the possible explanation for the different sweetnesses of the melon cultivars was discussed. Our findings improve the understanding of the molecular mechanisms determining sugar and organic acid accumulation and metabolism during oriental melon development and ripening.

## Data Availability Statement

The datasets presented in this study can be found in online repositories. The names of the repository/repositories and accession number(s) can be found below: https://www.ncbi.nlm.nih.gov/, PRJNA784415.

## Author Contributions

HC and TL conceived, designed the experiments, and wrote the manuscript. WK and TT performed the experiments. KR and KZ contributed to the material planting and sample collection. HC and HW contributed to the data analysis. All authors discussed the results, commented on the manuscript, read, and approved the final manuscript.

## Conflict of Interest

The authors declare that the research was conducted in the absence of any commercial or financial relationships that could be construed as a potential conflict of interest.

## Publisher’s Note

All claims expressed in this article are solely those of the authors and do not necessarily represent those of their affiliated organizations, or those of the publisher, the editors and the reviewers. Any product that may be evaluated in this article, or claim that may be made by its manufacturer, is not guaranteed or endorsed by the publisher.

## Footnotes

1
http://cucurbitgenomics.org/organism/3

## Supplementary Material

Supplementary Figure 1 PCA of metabolomics data from three developmental stages of melon cultivars A and B fruits. QC (quality control) represents a mixture of all fruit samples.

Supplementary Figure 2 PCA of the transcriptomics data from three developmental stages of melon cultivars A and B fruits.

Supplementary Figure 3 Venn diagrams of DEGs among different comparison groups.

Supplementary Table 1 Primer sequences for qRT-PCR analysis.

Supplementary Table 2 Total identified metabolites in the different ripening stages among oriental melon cultivars A and B fruits.

Supplementary Table 3 Extremely enriched KEGG pathways of DAMs among different comparison groups. The analysis criterion is *p* < 0.05.

Supplementary Table 4 Extremely enriched KEGG pathways of DEGs among different comparison groups. The analysis criterion is q-value ≤ 0.001.

Supplementary Table 5 Correlation statistics of DEGs and DAMs related to sugar and organic acids metabolism. The data were screened from the two comparison groups of AFS2 vs. BFS2 and AFS3 vs. BFS3.

Supplementary Table 6 The expression change in candidate genes related to soluble sugar and organic acid metabolism. The expression was based on the fold-change, and the data of BFS1 were used as the control.
