# Supplementary figures and images for: Identification of Key Gene Networks Controlling Soluble Sugar and Organic Acid Metabolism During Oriental Melon Fruit Development by Integrated Analysis of Metabolic and Transcriptomic Analyses
Source: Front Plant Sci. 2022 May 12;13:830517. doi: 10.3389/fpls.2022.830517 (PMC9135470; doi:10.3389/fpls.2022.830517)

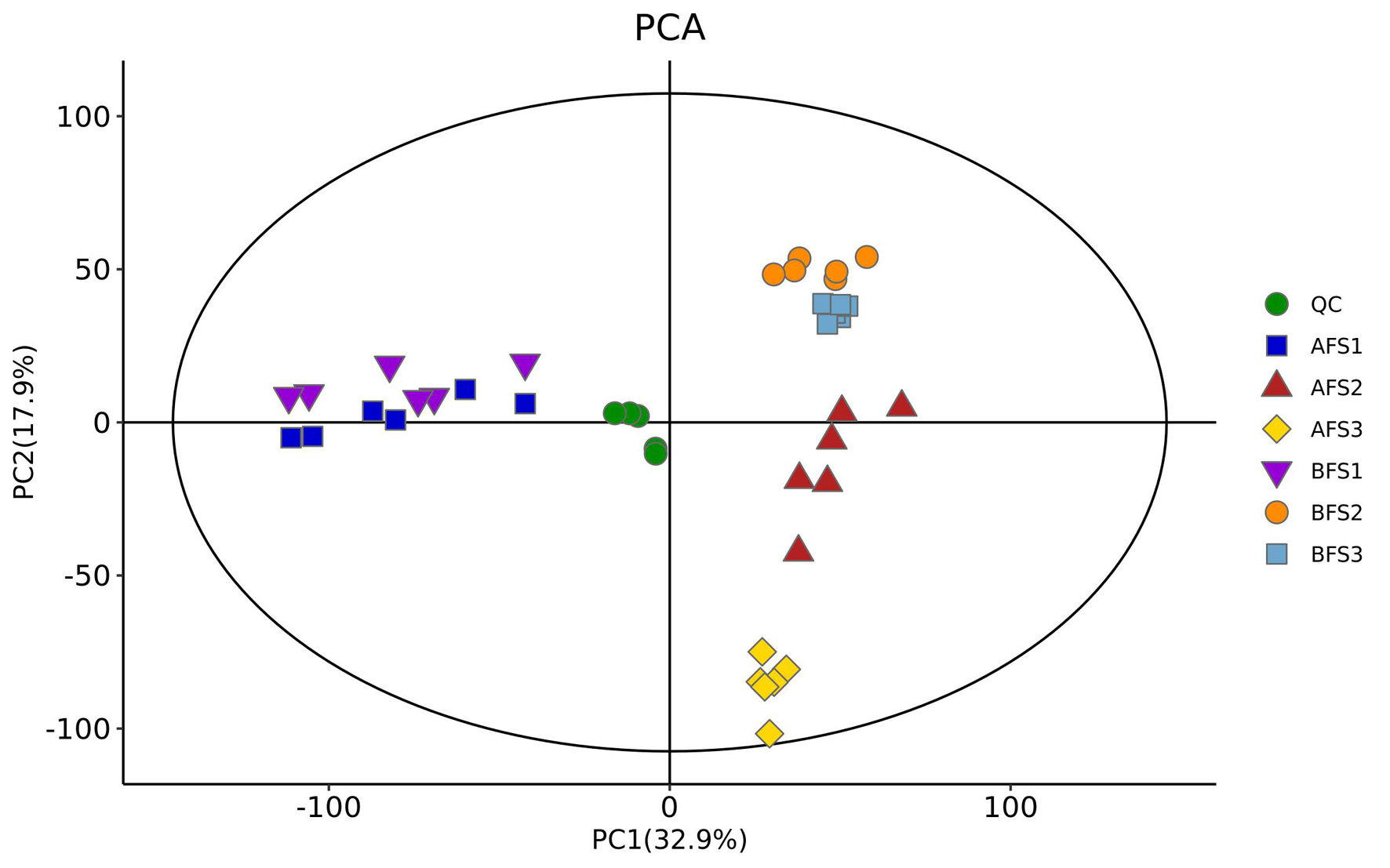

Supplement: Supplementary Figure 1 — PCA of metabolomics data from three developmental stages of melon cultivars A and B fruits. QC (quality control) represents a mixture of all fruit samples. [file Image_1.JPEG]

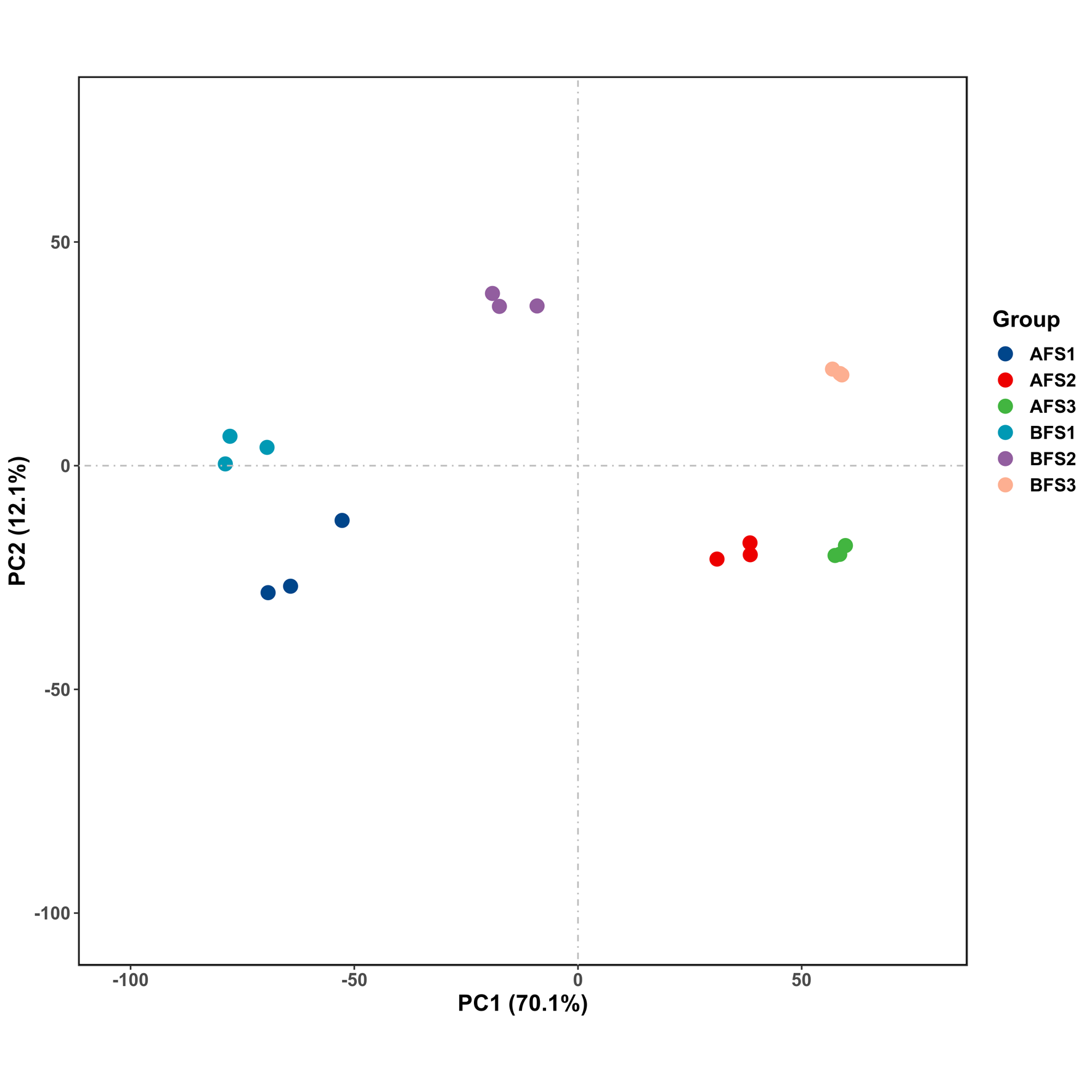

Supplement: Supplementary Figure 2 — PCA of the transcriptomics data from three developmental stages of melon cultivars A and B fruits. [file Image_2.PNG]

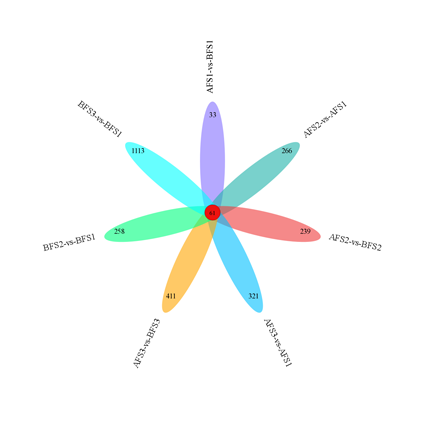

Supplement: Supplementary Figure 3 — Venn diagrams of DEGs among different comparison groups. [file Image_3.PNG]
