# Supplementary material for: Identification of Key Gene Networks Controlling Soluble Sugar and Organic Acid Metabolism During Oriental Melon Fruit Development by Integrated Analysis of Metabolic and Transcriptomic Analyses
Source: Front Plant Sci. 2022 May 12;13:830517. doi: 10.3389/fpls.2022.830517 (PMC9135470; doi:10.3389/fpls.2022.830517)
Supplement: Supplementary Table 1 — Primer sequences for qRT-PCR analysis. [file Table_1.DOCX]

**Supplementary Table 1** Primer sequences for qRT-PCR analysis.

| **Number** | **Gene** | **Gene ID** | **Forward primer** | **Reverse primer** | **Product length (bp)** |
| --- | --- | --- | --- | --- | --- |
| 1 | *RS-1* | MELO3C023110.2 | GGTGTTGTGGAAGGTCTCA | TCTGTTGCCAACCATCATCTAT | 82 |
| 2 | *RS-2* | MELO3C010314.2 | TCTGTTACTGGAGACGAAGG | AGGAAGAAACACGGTGTATATG | 88 |
| 3 | *CWINV3* | MELO3C010751.2 | ATGGGCCAATGTTCTACAAG | ATATTGAATGAGCCCACACC | 99 |
| 4 | *SS-2* | MELO3C025101.2 | GGGCAGATCAGTCTGTCTAT | AAGTTCTTCAATGGCAGGT | 80 |
| 5 | *SWEET* | MELO3C026184.2 | TTGCACTCTCATTCTTTCTCAC | GAACCCAACCACGTTTGGTA | 101 |
| 6 | *PDC1* | MELO3C009145.2 | TTCAGCCAAGAACTCCGA | GGCAGTAGAAATGGCAGTATC | 105 |
| 7 | *PK-2* | MELO3C006964.2 | AACAGAGGTTGGCTTTGTATC | AGTTAAGGCATCAGTAAAGGTC | 89 |
| 8 | *PDH-E1-3* | MELO3C022306.2 | GAATGTCCCATTTGAGGGC | TCAACATGAACACCAGGCA | 82 |
| 9 | *PDH-E1-4* | MELO3C002746.2 | CAAGGCTGATACTTCATCGG | CTCCAAACCTTCTCTTAGTGC | 85 |
| 10 | *ACO1-1* | MELO3C014437.2 | CAAAGGCTTAGAGGCTGC | GGGAGATGTTTGATTCAGGTAG | 92 |
| 11 | *IDH* | MELO3C025076.2 | CAGCTCATGGCACTGTTAC | CAAGCAAAGATGGACGCTAT | 88 |
| 12 | *NADP-ME* | MELO3C011129.2 | CTCTTAAACTTGTTGGAGGGAC | AAGCAATAAGCTCAGCTATACC | 93 |
| 13 | *GAPDH* |  | CTTTCTCTTCATCCCTTTGTTC | CTCTGCAAGGCGACTCTA | 116 |
